# Prognostic Value of Ki-67 in Breast Cancer Patients with Positive Axillary Lymph Nodes: A Retrospective Cohort Study

**DOI:** 10.1371/journal.pone.0087264

**Published:** 2014-02-03

**Authors:** Feng-yan Li, San-gang Wu, Juan Zhou, Jia-yuan Sun, Qin Lin, Huan-xin Lin, Xun-xing Guan, Zhen-yu He

**Affiliations:** 1 Sun Yat-sen University Cancer Center, State Key Laboratory of Oncology in South China, Department of Radiation Oncology, Collaborative Innovation Center of Cancer Medicine, Guangzhou, People's Republic of China; 2 Xiamen Cancer Center, Department of Radiation Oncology, the First Affiliated Hospital of Xiamen University, Xiamen, People's Republic of China; 3 Xiamen Cancer Center, Department of Obstetrics and Gynecology, the First Affiliated Hospital of Xiamen University, Xiamen, People's Republic of China; Okayama University, Japan

## Abstract

**Introduction:**

Ki-67 expression is a biomarker for proliferation. Its prognostic value is recognized in breast cancer (BC) patients with negative axillary nodes, but is less clear in BC patients with positive axillary lymph nodes.

**Methods:**

We retrospectively reviewed the medical records of 1131 Chinese BC patients treated from January 2002 to June 2007 and 450 patients met the inclusion criteria: positive nodes, adjuvant therapy, and complete biomarker profile (estrogen receptor (ER), progesterone receptor (PR), HER2, p53, Ki-67). Univariate and multivariate regression analysis were used to correlate biomarkers and tumor characteristics with metastasis free survival (MFS) and overall survival (OS).

**Results:**

Median follow-up time was 46 months (range 5–76 months). The Ki-67 expression was associated significantly with histological grade, ER, PR, HER2, and P53 status (P<0.05). Tumor stage, nodal stage, and ER status were independent prognostic factors for MFS. Ki-67 status was associated significantly with OS but not MFS. To determine whether the extent of LN involvement in the BC patients influenced the role of Ki-67 in survival rates, we compared these variables in patients with 1–3 positive lymph nodes (N1) to those of patients with ≥4 positive lymph nodes. Ki-67 status was an independent prognostic factor for MFS (Hazard Ratio, 3.27, P = 0.026) and overall survival (HR, 10.64, P = 0.007) in patients with 1–3 positive nodes (N1).

**Conclusions:**

The possibility that Ki-67 expression together with clinical factors can improve prediction of the prognosis of BC patients with 1∼3 positive axillary lymph nodes warrants further studies.

## Introduction

Development of comprehensive therapy has reduced the mortality rate of breast cancer patients [1]. However, regional and distant recurrences still threaten the lives of breast cancer patients [2], [3]. After therapy, any residual cancer cells that continue to proliferate can lead to a local recurrence and/or metastasis. Despite the significance of proliferation of residual breast cancer cells, most prognostic factors measure demographic characteristics of the patient (e.g. age), tumor status (e.g. grade, size, spread) or histological features (e.g. hormone receptor status, HER-2 status, and nodal status). Interest in a prognostic factor that measures proliferative status of breast cancer and predicts response to therapy is high [4]: the Ki-67 marker is a prominent candidate.

Ki-67 protein (also known as MKI67) is a cellular marker for proliferation. This nuclear protein is expressed in proliferating cells during G1 through M phases of the cell cycle, but is not detected in resting cells. The Ki-67 expression as detected by immunohistochemistry is one of the most reliable indicators of the proliferative status of cancer cells [5] and is referred to as Ki-67 henceforward. In 2009, at the St-Gallen breast cancer conference, Ki-67 was recommended as a biomarker for prognosis and sensitivity of cancer cells to endocrine therapy or chemotherapy [6]. In 2011, Ki-67 was regarded as one of the factors influencing molecular subtypes [7]. Ki-67 expression is closely associated with the growth and invasion of breast cancer: Ki-67-positive breast cancers are more active in growth, more aggressive in invasion, and more metastatic. Cheang et al. [8] (2009) integrated Ki-67 expression as a prognostic factor into molecular typing, and their results showed Luminal B breast cancer patients with positive axillary lymph nodes (ER and/or PR positive, HER-2 positive, ≥14% Ki-67 positive cells) had a poorer 10-year recurrence free survival rate (64% vs. 47%, P<0.001) and a poorer overall survival rate (74% vs. 59%, P<0.001) when compared with Luminal A breast cancer patients (ER and/or PR positive, HER2 negative, <14% Ki-67 positive cells). Furthermore, two meta-analyses showed that Ki-67 is an important factor affecting the recurrence of early breast cancer and the survival of breast cancer patients [9], [10]. The cut-off level for Ki-67 positive staining has varied from 5% to 30% [9], which complicates the comparison of the findings. The prognostic value of Ki-67 has been associated with poorer prognosis in breast cancer patients with negative axillary lymph nodes in most studies [11], [12], [13]. However, racial differences and ethnic origins appear to affect the frequency of high Ki-67 expression in breast cancer [14].

In Southern China, approximately 50% of breast cancer patients have 1 or more positive nodes at diagnosis [15]. Positive node status at diagnosis was associated significantly with lower rates of disease-free survival and overall survival [15]. Compared to studies of breast cancer patients with no positive nodes, the prognostic value of Ki-67 in breast cancer patients with positive axillary lymph nodes was investigated in fewer studies and was more variable [11]. Some studies observed a significant unfavorable prognostic value of Ki-67 [9]. Matsubara et al (2011) found that high Ki-67 expression in Japanese patients with breast cancer and positive axillary lymph nodes was an unfavorable prognostic factor for disease free survival (DFS) and overall survival [16]. Weisner et al (2009) observed a significantly higher Ki-67 overexpression in breast cancer tissues from Caucasian patients with 1–3 positive axillary nodes (N1) but not those with 4 or more positive nodes [17]. The role and the prognostic value of Ki-67 in breast cancer patients with positive axillary nodes are unknown.

In this retrospective study, the prognostic value of the Ki-67 marker was evaluated in Chinese patients with breast cancer and with one or more positive axillary lymph nodes.

## Patients and Methods

### Patient selection and treatments

This retrospective study was approved by the Institutional Review Board of Sun Yat-Sen University Cancer Center (Guangzhou, People's Republic of China). Written informed consent was obtained from the patients. The clinicopathological and demographic data in the medical reports of breast cancer patients with positive axillary lymph nodes who received surgical intervention at the Sun Yat-Sen University Cancer Center were retrospectively compiled. Inclusion criteria were women who: (1) had unilateral breast cancer, received mastectomy or breast-conserving surgery between January 2002 and June 2007, and showed one or more axillary lymph nodes positive for cancer cells by pathologic examination; (2) received standard adjuvant therapy after surgery according to the stages of breast cancer; (3) had no severe concomitant diseases; (4) had complete immunohistochemistry data including estrogen receptor (ER), progesterone receptor (PR), Her-2, Ki-67 and p53.

All BC patients included in the retrospective study had received chemotherapy with a median course of 6 cycles (range: 4–9 cycles). The CMF (cyclophosphamide, methotrexate, 5-fluorouracil) regimen was administered to 10 patients, and an anthracycline and/or taxane-containing regimen to 440 patients. Endocrine therapy was performed in 325 patients who were positive for ER and/or positive for PR. Tamoxifen was given to premenopausal patients, and postmenopausal were given either tamoxifen or aromatase inhibitors. Patients with ≥4 positive lymph nodes and T3-4 breast cancer patients receiving radical mastectomy received adjuvant radiotherapy at ipsilateral chest wall and supraclavicular/subclavicular areas (25 fractions of 2 Grey (Gy) for a total of 50 Gy). Patients who had undergone breast-preserving surgery received adjuvant radiotherapy of the whole breast and ipsilateral supraclavicular/subclavicular areas (25 fractions of 2 Gy) and with RT boost of the tumor bed (5 fractions of 2 Gy; total dose per patient was 60 Gy).

### Immunohistochemistry Determination of expressions of Ki-67 and p53

The immunohistochemistry staining methods for ER, PR, and HER2 were performed as recently described [18]. The expression levels of Ki-67 and p53 were determined by immunohistochemical analyses. Briefly, tissues were fixed in 10% formaldehyde for 24 h, routinely embedded in paraffin, and cut into 5-µm sections. Sections were adherent to APES coated slides and dried at 60°C for 2 h. Immunohistochemistry was performed according to the manufacturer's instructions, using Streptavidin-Peroxidase (SP-9000) kit, anti-Ki67 (ZM0166), P53 (ZM0408), C-erb2 (ZM0065), all from Zymed laboratories (San Francisco, CA, USA) with antigen retrieval performed according to the manufacturer's instruction. The slides were scored by counting the number of positive cells regardless of the staining intensity versus the total number of cells and calculating the percentage of positive cells (positive cells/total cells in one field), as previously described [19], the positivity of several fields were averaged and expressed as the ratio of positive cells per field to total cells per field: <10%, negative; 10%–25%: weakly positive; 26%–50%: positive; >50%: strong positive. A cut-off point of 25% was used to distinguish between the categories of low and high proliferative tumors, a value similar to 20% by Nishimura et al. [20] or >20% used by Kruger et al. [21] and Weisner et al. [17]. This retrospective study used the pathological reports included in the BC patients' records: the initial reports had been written by resident or attending pathologists and were confirmed by chief physician or pathology professor before submission.

### Follow-up and methods

Follow-up was performed by hospital visit, telephone or mail, and counted from the first day after surgery. The main end points were metastasis free survival (MFS) and overall survival (OS). Distant metastasis refers to the presence of breast cancer lesions at sites distant to the primary site. Different examinations were used to confirm potential metastases at distinct sites: bone metastasis required bone scan and MRI; lung metastasis usually was identified by repeated chest radiograph, followed by chest CT confirmation, or PET/CT confirmation; liver metastasis generally used ultrasound at follow up, and was followed by MRI or PET/CT if an abnormality were observed.

### Statistical analysis

The lymph node ratio was calculated for each individual as the ratio of the number of BC-containing lymph nodes divided by the total number of lymph nodes examined for the patient, as previously described [22]. The association between clinicopathologic factors and expression of Ki-67 was determined by using Chi-square/Fisher's exact tests. Data are expressed as number (percentage). Univariate and multivariate Cox proportional hazards analyses of distant metastasis-free survival and overall survival were performed to identify prognostic clinicopathologic factors for patients with axillary lymph node positive breast cancer. Variables with a significant association with MFS and OS in univariate analysis (*P*-value <0.05) were selected and evaluated by multivariate analysis. All statistical assessments were 2-sided, and statistical significance was set at *P*<0.05. Statistical analyses were performed with SPSS 15.0 Statistics Software (SPSS Inc.). This study had 99.4% power at alpha = 5% to detect a HR of 2.07 among women with 4+ positive LN.

## Results

The medical records of 1131 female breast cancer patients who were treated in Sun Yat-sen University Cancer Center from January 2002 to June 2007 were reviewed and analyzed. The 450 female patients who met the inclusion criteria had a mean age of 47.3 years (ranged from 23 to 81 years). The median number of axillary lymph nodes removed was 16 (range: 5 to 73) and the median lymph node ratio (LNR) was 0.18. According to the TNM classification developed by UICC, most patients were diagnosed with stage II breast cancer (n = 224; 49.8%) or stage III breast cancer (n = 206; 45.8%). Sixteen of 450 patients (3.6%) received breast preserving surgery. Pathological examination showed invasive ductal carcinoma in 420 patients (93.3%), invasive lobular carcinoma in 10 (2.2%), medullary carcinoma in 6 (1.3%), endocrine carcinoma in 5 (1.1%), mucinous adenocarcinoma in 5 (1.1%) and mixed carcinoma in 4 (0.9%).

Most patients were premenopausal (63.6%), had T1–T2 stage cancer (83.1%) (Table 1) and had received an anthracycline and/or taxane-containing chemotherapy regimen (n = 440; 97.8%). Immunohistochemistry revealed that 290 patients were positive for ER, 312 positive for PR, 106 patients were positive for HER2 (3+), and 199 were positive for p53 (Table 1). The Ki-67 staining were scored as negative (<10%), weakly positive (<25%), positive (25%–50%), and strongly positive (>50%) in 39, 214, 122, and 75 breast cancer patients, respectively. To obtain similar sized groups for association studies, the 450 breast cancer samples were categorized into two groups: patients with breast tumors with weakly positive Ki-67 staining (<25% Ki-67 positive cells, n = 253, 56.2%) or patients with breast tumors with positive Ki-67 staining (≥25% positive cells, n = 197, 43.8%), similar to cutoff values of ≥20% in previous studies [17], [20], [21]. Table 1 shows the association between Ki-67 and clinicopathologic characteristics. Histological grade, ER, PR, HER2 and P53 status were significantly associated with expression of Ki-67 (P<0.05).

**Table 1:** The clinicopathologic characteristics of entire cohort and association between Ki-67 and clinicopathologic characteristics.

Characteristic		Ki-67		
	Total (n = 450)	≤25% positive (n = 253)	>25% positive (n = 197)	P-value
Age, n (%)				
<35 years	43 (9.6)	28 (11.1)	15 (7.6)	0.216
≥35 years	407 (90.4)	225 (88.9)	182 (92.4)	
Menopausal status, n (%)				
Premenopausal	286 (63.6)	163 (64.4)	123 (62.4)	0.663
Postmenopausal	164 (36.4)	90 (35.6)	74 (37.6)	
Tumor stage, n (%)^a^				
T1	89 (19.8)	59 (23.3)	30 (15.2)	0.113
T2	285 (63.3)	152 (60.1)	133 (67.5)	
T3	46 (10.2)	23 (9.1)	23 (11.7)	
T4	30 (6.7)	19 (7.5)	11 (5.6)	
Nodal stage, n (%)				
N1 (1–3)	262 (58.2)	142 (56.1)	120 (60.9)	0.251
N2 (4–9)	99 (22.0)	54 (21.3)	45 (22.8)	
N3 (≥10)	89 (19.8)	57 (22.5)	32 (16.2)	
Histological grade, n (%)				
I	8 (1.8)	7 (2.8)	1 (0.5)	0.010*
II	151 (33.6)	87 (34.4)	64 (32.5)	
III	99 (22.0)	43 (17.0)	56 (28.4)	
IV	192 (42.7)	116 (45.8)	76 (38.6)	
ER status, n (%)				
Negative	160 (35.6)	72 (28.5)	88 (44.7)	<0.001*
Positive	290 (64.4)	181 (71.5)	109 (55.3)	
PR status, n (%)				
Negative	138 (30.7)	66 (26.1)	72 (36.5)	0.017*
Positive	312 (69.3)	187 (73.9)	125 (63.5)	
HER-2 status, n (%)				
Negative	344 (76.4)	210 (83.0)	134 (68.0)	<0.001*
Positive	106 (23.6)	43 (17.0)	63 (32.0)	
P53 status, n (%)				
≤25% positive	251 (55.8)	176 (69.6)	75 (38.1)	<0.001*
>25% positive	199 (44.2)	77 (30.4)	122 (61.9)	
Treatments				
Chemotherapy				0.688
CMF	10 (2.2)	5 (2.0)	5 (2.5)	
Anthracycline &/or taxane	440 (97.8)	248 (98.0)	192 (97.5)	
Radiotherapy	262 (58.2)	142 (56.1)	120 (60.9)	0.307
Endocrine therapy	104 (23.1)	46 (18.2)	58 (29.4)	0.005*

a Tumor stages: T1, tumor nodule is less than 2 cm; T2, tumor size is between 2 cm and 5 cm; T3, tumor size is larger than 5 cm; T4, tumor has grown through skin or chest wall.

* Significant difference between percentage of samples with Ki-67 expression ≤25% positive versus those with >25% positivity by using chi-square/Fisher's exact test.

ER, estrogen receptor; PR, progesterone receptor.

During the follow-up period, 41 patients died. The median follow-up time was 46 months (range 5 to 76 months). The 5-year overall survival and metastasis-free survival rates were 88.1% and 81.9%, respectively. The 5-year overall survival rate of weakly stained Ki-67 breast cancer patients (≤25%) was significantly greater (93%) than the OS rate of the Ki-67 positive (>25%) breast cancer patients (82%) (Log-rank P = 0.022). The 5-yr metastasis-free survival rates of patients with weakly stained Ki-67 breast tumor tissues (≤25%) and patients with Ki-67 positive breast tumor tissues (>25%) were 85% and 77.1% (Log-rank P = 0.334), respectively.

The univariate Cox proportional hazard regression model indicated the following prognostic factors for metastasis-free survival: tumor stage, nodal stage, ER status, HER-2 status, and neo-adjuvant chemo-therapy, radiotherapy, endocrine therapy, (*P*<0.05). The multivariate Cox proportional hazard analysis of these factors showed that the tumor stage (hazard ratio (HR), 3.40, 95% C.I., 2.08–5.55; P<0.001), nodal stage (HR, 3.20, 95% C.I., 1.56–5.50; P<0.001) and ER status (HR, 2.24, 95% C.I., 1.33–3.77; P = 0.002) were independent prognostic factors for metastasis-free survival in patients with axillary lymph node positive breast cancer.

The prognostic factors for overall survival was determined by univariate and multivariate Cox proportional hazard regression analysis. The univariate Cox proportional hazard regression model indicated tumor stage, nodal stage, ER, PR, HER2, radiotherapy, endocrine therapy, and Ki-67 status (*P*<0.05) as prognostic factors. Table 2 revealed that the multivariate Cox proportional hazard analysis revealed the Ki-67 status was associated significantly with overall survival but not metastasis-free survival in patients with axillary lymph node positive breast cancer. (Table 2)

**Table 2:** Cox proportional hazards regression analysis of potential prognostic factors for Metastasis-free survival and overall survival.

	metastasis-free survival						overall survival					
	Univariate^a^			Multivariate^a^			Univariate^a^			Multivariate^a^		
	HR^b^	95%CI^b^	P-value	HR^b^	95%CI^b^	P-value	HR^b^	95%CI^b^	P-value	HR^b^	95%CI^b^	P-value
**Node status Ki-67^+^ >25% vs. Ki-67^+^ ≤25%**												
Overall (n = 450)	1.26	(0.79, 2.01)	0.335	N/A			2.07	(1.01, 3.90)	0.024*	2.07	(1.08, 3.95)	0.028*
Patients with 1–3 positive lymph nodes (n = 262)	3.71	(1.34, 10.27)	0.012*	3.27	(1.16, 9.27)	0.026*	12.06	(2.37, 61.41)	0.003*	10.64	(1.90, 59.50)	0.007*
Patients ≥4 positive lymph nodes (n = 188)	0.91	(0.52, 1.60)	0.747	N/A			1.3	(0.61, 2.76)	0.500	NA		
**Chemotherapy**				3.99	(1.45,11.0)	0.007*				NA		
**Endocrine therapy**				1.71	1.05,2.80	0.032				2.58	(1.39, 4.79)	0.003*
**Radiotherapy**				0.24	(0.15,0.41)	0.001*				0.342	(0.18, 0.65)	0.001*

a The main predictor of interest Ki-67 was used in univariate model alone; Ki-67 with other covariates, tumor stage, nodal stage, histological grade, ER status, HER-2 status, P53 status, neo-adjuvant therapy, radiotherapy, and endocrine therapy were included in the model.

b Abbreviations: HR, hazard ratio; CI, confidence interval; N/A, not available.

* Significant factor, P<0.05.

To determine whether the extent of LN involvement in the breast cancer patients influenced the role of Ki-67 in survival rates, we compared these variables in patients with 1–3 positive lymph nodes (n = 262) to those of patients with more than 4 positive lymph nodes (n = 188). Power calculations indicated that detection of difference in the survival rates (MFS or OS) at the 5% significance level would require a sample size of 77 and 111 for BC patients with 1–3 positive lymph nodes and BC patients with ≥4 positive lymph nodes, respectively. Based on the observed median survival time of 42.67 months in 111 patients with 1–3 positive lymph nodes and 45.80 months in 77 BC patients with ≥4 positive lymph nodes with a Hazard ratio 2.07, the observed power was derived with equivalent to 99.4% under the 5% significance level. Table 2 also shows the results of Cox proportional hazards regression analysis of potential prognostic factors for metastasis-free survival and overall survival in patients with 1–3 positive lymph nodes versus patients with more than 4 positive lymph nodes. According to multivariate Cox proportional hazard analysis, Ki-67 status was an independent prognostic factor for metastasis-free survival (HR, 3.27, 95% C.I., 1.16–9.27; P = 0.026) and overall survival (HR, 10.64, 95% C.I., 1.90–59.50; P = 0.007) in patients with 1–3 positive lymph nodes breast cancer. Tumor stage and ER status, but not Ki-67, were significant prognostic factors for MFS and OS in patients with ≥4 positive lymph nodes according to univariate and multivariate analysis (Table 2).

## Discussion

Approximately half of breast cancer patients in China have one or more positive lymph nodes [15] and these patients show significantly lower rates of disease-free survival and overall survival [15] than breast cancer patients with only negative nodes. Despite post-operative adjuvant therapy based on the pathology, status of axillary lymph nodes, tumor size and status of hormone receptors in breast cancer patients [2], [23], more than 15% of patients develop incurable disease [3]. Identification of these patients with non-responsive breast cancer so their therapy can be individualized is a hot topic in breast cancer research. Biomarker profiles (estrogen receptor, progesterone receptor, HER2, triple negative) can provide prognostic value for probability of therapeutic responses, especially to targeted therapies. Ki-67 is a marker reflecting the proliferative capability of cancer cells and is being widely investigated in breast cancer studies [4], [24], [25].

Our results indicate that the prognostic value of Ki-67 is influenced by the number of positive lymph nodes of the breast cancer patients. Breast cancer patients with 1–3 positive axillary lymph nodes and >25% Ki-67 positive cells had significantly worse MFS and OS, in agreement with three reports [9], [16], [17]. High Ki-67 (≥10%) in breast cancer patients with moderate risk for recurrence (including those with 1–3 positive axillary lymph nodes) was an independent prognostic factor for shorter disease-free survival and overall survival [26], but a cutoff value of 10% in our study was not significantly associated with metastases-free survival or overall survival (Table S1). However, the size of the ≥10% Ki-67 staining group (n = 411) in our study was much greater than the size of the <10% Ki-67 staining group (n = 39). The small size of the <10% Ki-67 staining group (n = 39) greatly reduced the statistical power of the analysis and likely contributed to finding the results insignificant. Wiesner et al. [17] and meta-analysis [9] also support the prognostic value of high Ki-67 expression that influences the overall survival of early breast cancer patients (N0-1). The above findings suggest that high Ki-67 expression may serve as a prognostic risk factor in patients with 1–3 positive axillary lymph nodes and is associated with a poorer prognosis, thus indicating a need for individualized therapy.

Ki-67 expression is also a prognostic factor for the survival of patients with ≥4 positive axillary lymph nodes in a study by Crabb et al [27]. In comparison, Ki-67 tumor expression in patients with high risk (>4 positive axillary lymph nodes) was not an independent prognostic factor in our study nor in two additional studies [16], [28]. The disparate results may arise from the following potential reasons: (1) breast cancer patients with ≥4 positive axillary lymph nodes have a higher cancer load, and the status of ≥4 axillary lymph nodes itself is a high risk factor for a poor prognosis [29]. (2) One major difference in the patient populations was the administration of adjuvant therapy post-surgery: Approximately half of patients in the Crabb et al.'s study did not receive any adjunct chemotherapy and 22.4% received no adjunct radiotherapy [27]. In the present study, our patients with ≥4 positive axillary lymph nodes received radiotherapy after surgery, which may benefit patients by inhibiting the proliferation of residual cancer cells. Because head and neck cancers with high Ki-67 expression were more sensitive to radiotherapy [30], it is feasible that radiotherapy improved the survival of this subgroup of breast cancer patients and overcame the negative influence of high Ki-67. However, further studies that investigate the influence of Ki-67 expression on the sensitivity of breast cancer to radiotherapy are warranted. In most studies [8], the majority of patients were also treated with adjuvant therapy with reduced intensity. Thus, the insufficient adjunct therapy may influence the determination of prognosis with Ki-67 expression.

Currently, most studies support the positive correlation between status of axillary lymph nodes and high Ki-67 expression, but the association of Ki-67 expression with the number of metastatic lymph nodes is not universal. In the present study, our results showed the Ki-67 expression was not related to the number of metastatic lymph nodes, in agreement with a previous report [31]. In addition, Ki-67 expression in axillary lymph nodes is significantly higher in lymph nodes than that in primary cancer in some cases [31], [32], which may be explained that cancer cells with potent proliferative capability are more likely to metastasize from the primary cancer to the axillary lymph nodes. Analysis of patients with divergent Ki-67 expression in primary versus metastatic lesions suggests that the prognostic value of Ki-67 (>10%) on survival was greater in the tumors located in the nodes than that in the primary lesion [24], [31].

Potential limitations of this manuscript include the variation in quantitating Ki-67 staining by immunohistochemistry between the two pathologists. Despite the potential variability of the quantitation of Ki-67 IHC staining, statistical analysis indicated that Ki-67 status was an independent prognostic factor for overall survival (P = 0.007) and MFS (P = 0.026) in patients with 1–3 positive nodes (N1).

To date, several studies have also indicated that Ki-67 expression may be a marker for predicting the sensitivity of chemotherapy, and that Ki-67 expression before and after neoadjuvant therapy may be employed for predict prognosis [33], [34]. Currently, differences in the Ki-67-detection methods and cutoff points likely contribute to the varied conclusions on the prognostic value of Ki-67 expression in breast cancer patients. Thus, prospective studies with large sample size, standardized methodology and specialized personnel for Ki-67 detection in a single center or international guidelines are warranted [4], [35].

Taken together, Ki-67 expression together with clinical factors may favorably predict the prognosis of breast cancer patients with positive axillary lymph nodes, especially for those with 1–3 positive axillary lymph nodes, which may provide reference for prescribing individualized therapy of breast cancer.

## Supporting Information

Table S1 Cox proportional hazards regression analysis of potential prognostic factors for Metastasis-free survival and overall survival. (Based on different cut-off values of Ki67 expression).
(DOC)
