# Supplementary material for: Prognostic Value of Ki-67 in Breast Cancer Patients with Positive Axillary Lymph Nodes: A Retrospective Cohort Study
Source: PLoS One. 2014 Feb 3;9(2):e87264. doi: 10.1371/journal.pone.0087264 (PMC3911937; doi:10.1371/journal.pone.0087264)
Supplement: Table S1 — Cox proportional hazards regression analysis of potential prognostic factors for Metastasis-free survival and overall survival. (Based on different cut-off values of Ki67 expression). (DOC) [file pone.0087264.s001.doc]

Table S1. Cox proportional hazards regression analysis of potential prognostic factors for Metastasis-free survival and overall survival. (Based on different cut-off values of Ki67 expression).

|  | metastasis-free survival | | | | | | |  | overall survival | | | | | | |
| --- | --- | --- | --- | --- | --- | --- | --- | --- | --- | --- | --- | --- | --- | --- | --- |
|  | Univariate^a^ | | |  | Multivariate^a^ | | |  | Univariate^a^ | | |  | Multivariate^a^ | | |
|  | HR^b^ | 95%CI^b^ | P-value |  | HR^b^ | 95%CI^b^ | P-value |  | HR^b^ | 95%CI^b^ | P-value |  | HR^b^ | 95%CI^b^ | P-value |
| **Node status** |  |  |  |  |  |  |  |  |  |  |  |  |  |  |  |
| **Ki-67^+^>25% (n=253) vs. Ki-67+ ≤25% (n=197)** |  |  |  |  |  |  |  |  |  |  |  |  |  |  |  |
| Overall (n=450) | 1.26 | (0.79, 2.01) | 0.335 |  | N/A |  |  |  | 2.07 | (1.01, 3.90) | 0.024* |  | 2.07 | (1.08 , 3.95) | 0.028* |
| Patients with 1-3 positive lymph nodes (n=262) | 3.71 | (1.34, 10.27) | 0.012* |  | 3.27 | (1.16 , 9.27) | 0.026* |  | 12.06 | (2.37, 61.41) | 0.003* |  | 10.64 | (1.90 , 59.50) | 0.007* |
| Patients ≥ 4 positive lymph nodes (n=188) | 0.91 | (0.52, 1.60) | 0.747 |  | N/A |  |  |  | 1.3 | (0.61, 2.76) | 0.5 |  | N/A |  |  |
|  |  |  |  |  |  |  |  |  |  |  |  |  |  |  |  |
| **Node status** |  |  |  |  |  |  |  |  |  |  |  |  |  |  |  |
| **Ki-67^+^>10% (n=411) vs. Ki-67+ ≤10%(n=39)** |  |  |  |  |  |  |  |  |  |  |  |  |  |  |  |
| Overall (n=450) | 1.13 | (0.49 , 2.64) | 0.769 |  | N/A |  |  |  | 1.077 | (0.38 , 3.06) | 0.889 |  | 0.88 | (0.30 , 2.59) | 0.820 |
| Patients with 1-3 positive lymph nodes (n=262) | 2.39 | (0.32 , 17.95) | 0.396 |  | 2.1 | (0.28 , 16.09) | 0.474 |  | 2.66 | (0.33 , 21.67) | 0.36 |  | 3.42 | (0.41 , 28.83) | 0.259 |
| Patients ≥ 4 positive lymph nodes (n=188) | 0.677 | (0.27 , 1.72) | 0.412 |  | N/A |  |  |  | 0.6 | (0.18 , 2.00) | 0.408 |  | N/A |  |  |
